# Randomized controlled clinical trial comparing the efficacy and tolerability of aripiprazole and sodium valproate in the treatment of Tourette syndrome

**DOI:** 10.1186/s12991-019-0245-3

**Published:** 2019-10-10

**Authors:** Deshuang Tao, Tangwu Zhong, Shuxia Ma, Jialin Li, Xiaojie Li

**Affiliations:** 1 Jiamusi University, Jiamusi, 154002 Heilongjiang China; 2 College of Rehab Medicine, Jiamusi University, Jiamusi, China; 3 Rehab Center for Child CP, Jiamusi, Heilongjiang China; 4 Institute of Pediatric Neurological Disorders, Jiamusi University, Jiamusi, China; 5 Jiamusi Central Hospital, Jiamusi, China

**Keywords:** Aripiprazole, Sodium valproate, Treating, Tourette syndrome, Clinical trial

## Abstract

**Objective:**

This study compared the efficacy and tolerability of sodium valproate and aripiprazole in the treatment of Tourette syndrome (TS).

**Method:**

24 children and adolescents with a diagnosis of TS from the Jiamusi Central Hospital between January 2014 and August 2017 were randomly divided into sodium valproate group and aripiprazole group according to the order of clinic visits and treated for 10 days. Tic severity was rated using the Yale Global Tic Severity Scale (YGTSS) and the Clinical Global Impressions Scale for tics (CGI-Tics) and the adverse reactions were valued using the Treatment Emergent Symptom Scale (TESS) at baseline and at each follow-up visit.

**Results:**

The TTS score in the YGTSS scale decreased over time in both groups while the aripiprazole group was significantly higher on the 5th day (*p* < 0.05) and 10th day (*p* < 0.05) than the sodium valproate group. There was no significant difference in TESS score between the two groups.

**Conclusions:**

The study indicates that the patients treated with sodium valproate injection have a faster onset time than the patients treated with oral aripiprazole in controlling tics.

## Introduction

Tourette syndrome (TS), also known as Gilles de la Tourette syndrome, is a childhood-onset condition characterized by chronic motor and vocal tics. It has been estimated that about 1% of school-age children have TS [1]. But unfortunately the exact mechanisms have not been elucidated. Family studies [2] show that the incidence of TS in first-degree relatives of TS patients is 10–100 times higher than that of the general population. Probably the most accepted current hypothesis regarding the underlying pathophysiology and pathological anatomy of TS is that there is an impairment of cortical inhibition of motor programs that are spontaneously generated in the basal ganglia and expressed. Available evidence also supports involvement of cortico–striatal–thalamo–cortical (CSTC) circuits and their interconnecting brain regions in the pathophysiology of tics [3]. Several neurotransmitters, including dopamine, GABA and glutamate, play an important role within CSTC circuitry and have been proposed to have roles in both habitual behavior formation and in the pathophysiology of tics [4–8]. Multiple neurotransmitter abnormalities and their signaling may also lead to dysfunction of the CSTC neural network and subsequent clinical manifestations of TS. The pathogenesis of TS is also associated with excessive neurotrophic effects and pro-toxic effects of excitatory amino acids (EAA) in the brain [9]. Astrocyte-specific induction of knockout of EAAT2 in mice has been shown to result in pathological repetitive self-modification and twitch-like head shaking associated with glutamate hyperexcitability [10]. Short-term sodium valproate treatment augmented EAAT1 translocation to the cell membrane, whereas prolonged or chronic sodium valproate treatment resulted in an upregulation of EAAT1 mRNA and protein levels, as well as glutamate transport and production of glutamine. The treatment of TS involves appropriate education and support. Tics can be treated with habit reversal cognitive behavioral therapy [11], pharmacotherapy [12], local intramuscular injections of botulinum toxin [13] and some severe, refractory cases have responded to deep brain stimulation surgery (DBS) [14]. However, the cost of treatment and the risk of surgery still restrict its widespread development. How to control tics quickly for people whose condition seriously affects the daily life is very important.

The atypical antipsychotic aripiprazole is a dopamine D2- and serotonin 5-hydroxytryptamine (5-HT)1A receptor partial agonist and 5-HT2A receptor antagonist [15] that is approved by the United States and South Korea for the treatment of tic disorder. Generally, pediatric patients whose oral aripiprazole at doses of 5-, 10-, 15-, or 30 mg/day and adjusted according to the efficacy and side effects. Sodium valproate controls tics by regulating central GABA system function including enhancing gamma-aminobutyric acid (GABA) synthesis and inhibiting GABA degradation and directly stimulating GABA-A receptors to increase GABA levels and up-regulate excitatory amino acid transporter (EAAT)1 mRNA and protein levels and sodium valproate injection is the first line of medication to control the status of epilepsy. In order to understand the efficacy of sodium valproate injection in controlling the patients with a diagnosis of TS, our department selected 24 patients with acute severe tic and compared with oral aripiprazole.

### Participant characteristics

Eligible patients were aged 6–16 years (20 males, 4 females; mean ± SD age = 9.9 ± 3.3 years) with a diagnosis of TS (Diagnostic and Statistical Manual of Mental Disorders, Fourth Edition, Text Revision [DSM-IV-TR]) (American Psychiatric Association 2000), confirmed by the Kiddie Schedule for Affective Disorders and Schizophrenia—Present and Lifetime Version; with a Yale Global Tic Severity Scale Total Tic Score (YGTSS-TTS) ≥ 30 at screening were recruited at Jiamusi Central Hospital from January 2014 to August 2017. Before determining eligibility, we obtained informed consent from both the study subject and the child’s primary caregiver. The study protocol was approved by our local Institutional Review Board. Each patient was subsequently examined by a board-certified child psychiatrist. However, patients with any of the following characteristics were excluded from the study: included current psychotic symptoms. Subjects with an IQ ≤ 70 on the Wechsler Intelligence Scale for Children-Revised (WISC-R), as were patients with previous or current seizure episodes, electroencephalogram (EEG) abnormalities, and those who had used aripiprazole and sodium valproate previously, people who were hypersensitive to sodium valproate or aripiprazole were also excluded, and patients had no significant abnormalities in laboratory results, including serum chemistries, hematology, urinalysis.

### Study design

All subjects were evaluated at baseline by routine laboratory tests, electrocardiogram (ECG), resting pulse rate and blood pressure while sitting, height and weight measurement, medical history, and physical and neurological examinations. The patients were assigned by a randomization within our hospital. Briefly, after the patients passed the inclusion/exclusion criteria and gave consent for the study, they were numbered serially at our hospital by one “special doctor” according to the order of visits and then we use the random number table for grouping. The odd number selects sodium valproate, the even number chooses aripiprazole. The “special doctor” was not involved in the grouping and treatment. Patients in the aripiprazole group started at a dose of 2.5 mg/day if weight < 50 kg, which was increased to 5.0 mg/day 5 days later; Patients started at a dose of 5 mg/day if weight ≥ 50 kg, which was increased to 10.0 mg/day 5 days later in the aripiprazole group. The sodium valproate was dissolved in 20 ml saline (0.9% NaCl) at a dose of 15 mg/kg within 5 min and given intravenously once every 12 h. Patients were assessed every 5 days.

### Measurements

Symptom severity was assessed by the principal investigator. The primary efficacy endpoint was the YGTSS mean change (including YGTSS-motor scores, YGTSS-phonic scores, YGTSS-total scores, YGTSS-impairment scores) from pretreatment baseline to 10 days. The YGTSS [16–20] is a semi-structured clinical interview designed to assess current tic severity; this scale yields three summary scores; total motor (0–25), total phonic (0–25), and total tic (sum of motor and phonic) scores. The YGTSS also contains an impairment scale (0–50), which evaluates the global level of functional impairment arising from tics. Because this study was designed to compare not only efficacy in reducing tic symptoms, but also the global level of functional impairment, so the primary outcome measure contains YGTSS-motor scores, YGTSS-phonic scores, YGTSS-total tic scores, and YGTSS-impairment scores. The YGTSS was administered to each subject at each visit. Secondary outcome measures included the Clinical Global Impressions-Improvement scale [21] (CGI-I), Scores of 1 (very much improved) or 2 (much improved) in the CGI-I were regarded as positive responses. Adverse effects associated with these drugs were assessed using the Treatment Emergent Symptom Scale (TESS) [22], which included the most commonly encountered side effects of aripiprazole and sodium valproate, as well as general questions on health issues, current illnesses or injuries, and concomitant medical treatments. At the study endpoint, physical and neurological examinations, laboratory tests, and ECG assessments were repeated. All tests were admitted by a single psychiatrist, who was blinded to dose changes and the drug of choice.

### Statistical analyses

**Fig. 1:**
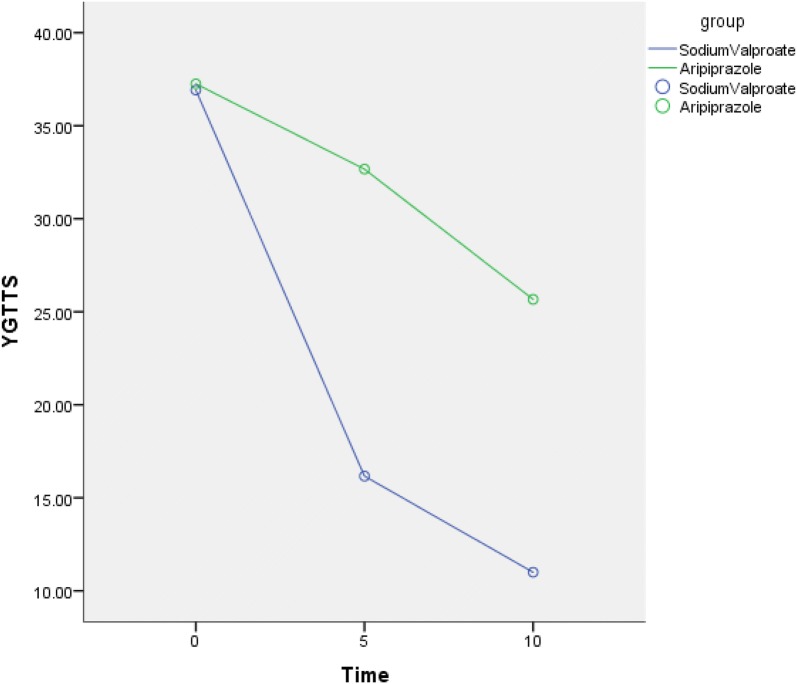
The mean YGTSS Total Tic scores from baseline to endpoint

Analyses were performed using SPSS 24. The demographic and clinical characteristics of samples are described in terms of means, standard deviations, range, and proportions as needed. Independent sample *t* test and repeated measures analysis of variance was applied when the measurement data would follow a normal distribution or nonparametric statistics was applied where the measurement data are not normal distribution. Count data using *χ*^2^ test. This study was an open-label design, with each subject contributing baseline and post-treatment measures. Significance is judged at level *p* = 0.05, two-sided (Fig. 1).

## Results

### Participant characteristics

Our hospital diagnosed and treated 723 tic disorder patients from 2014 to 2017, of which 248 patients had simple tic symptoms and the YGTSS scores ranged from 4 to 6, so they had no drug treatment but were given health guidance. 438 patients scored less than 25, the patient chose oral small doses of aripiprazole or clonidine transdermal patches or other Chinese medicine treatment. 37 people met the criteria for inclusion in this experiment, and 24 (64.9%) agreed to participate in the treatment. Of the 24 children and adolescents with Tourette syndrome, 12 were prescribed aripiprazole and 12 were prescribed sodium valproate. The age range is 6–16 years and the average age is 9.96. The mean duration of illness was 2.77 ± 0.92 years. The gender ratio, total IQ, comorbid conditions, duration of illness, and study medications did not differ between the two groups (*p* > 0.05). Twelve participants (50%) had other comorbid psychiatric disorders, the most common being anxiety disorder (20.83%) (Table 1).

**Table 1:** Demographic and clinical characteristics of the 24 children and adolescents with TS

	Sodium valproate (*n* = 12)	Aripiprazole (*n* = 12)	*χ*^2^/*t*/*Z*	*p*
Sex			0.000	> 0.999
Male	10	10		
Female	2	2		
Age	9.75 ± 3.42	10.17 ± 3.43	0.298	0.768
Weight	34.75 ± 9.88	34.67 ± 7.79	0.023	0.982
Duration of tic disorders	3.00 (2.00, 3.38)	2.75 (2.00, 3.78)	0.060	0.977
IQ	96.08 ± 6.83	97.17 ± 7.93	0.359	0.723
Comorbidities, *n*%				
ADHD	3, 25%	1, 8.33%		
ODD	1, 8.33%	1, 8.33%		
OCD	1, 8.33%	0		
Emotional disorder	216.67%	2, 16.67%		
Anxiety disorder	5, 41.67%	2, 16.67%		
Depressive disorder	0	1, 8.33%		

### Efficacy of aripiprazole and sodium valproate: Yale Global Tic Severity Scale

**Fig. 2:**
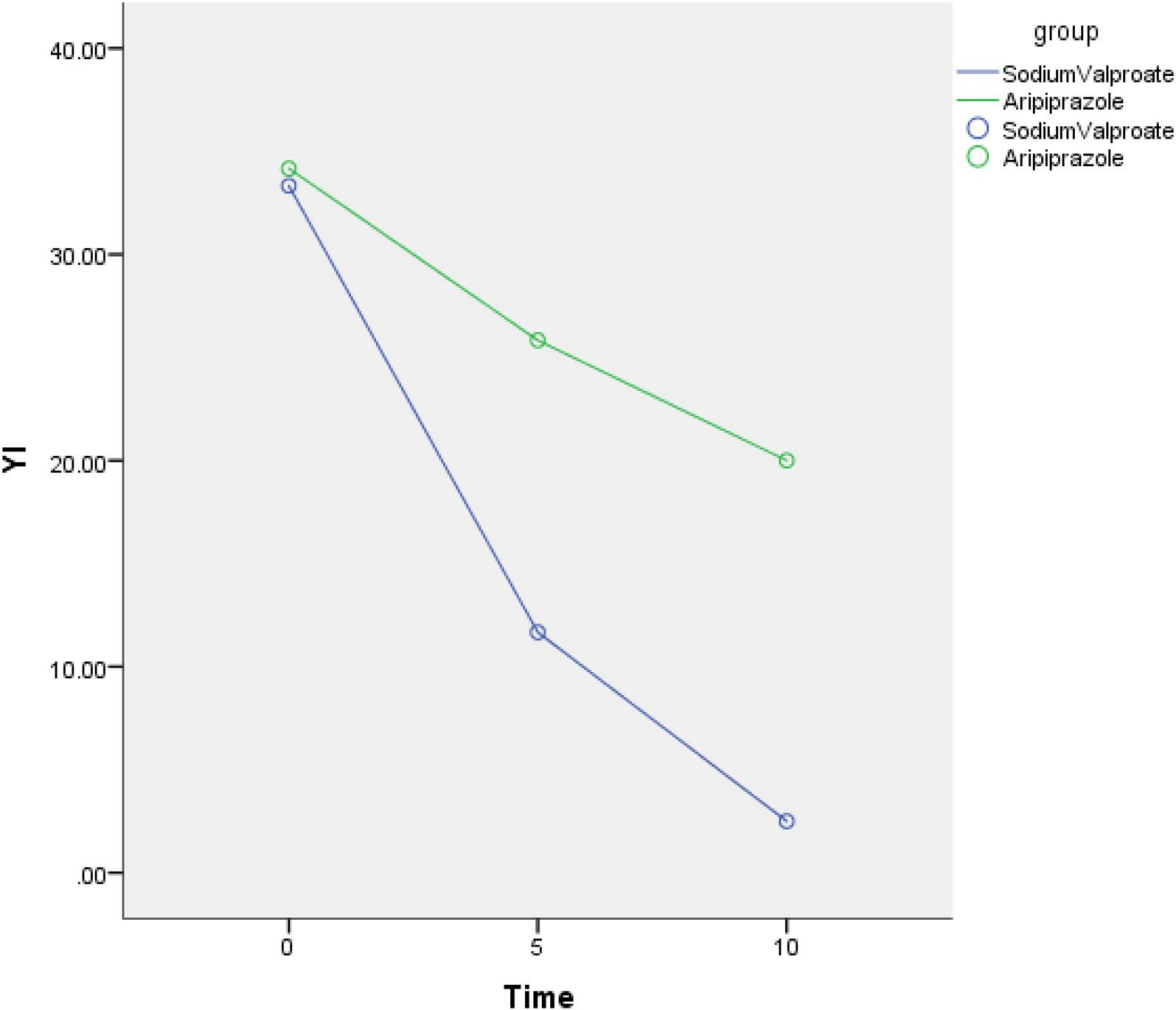
The mean YGTSS impairment scale scores from baseline to endpoint

**Fig. 3:**
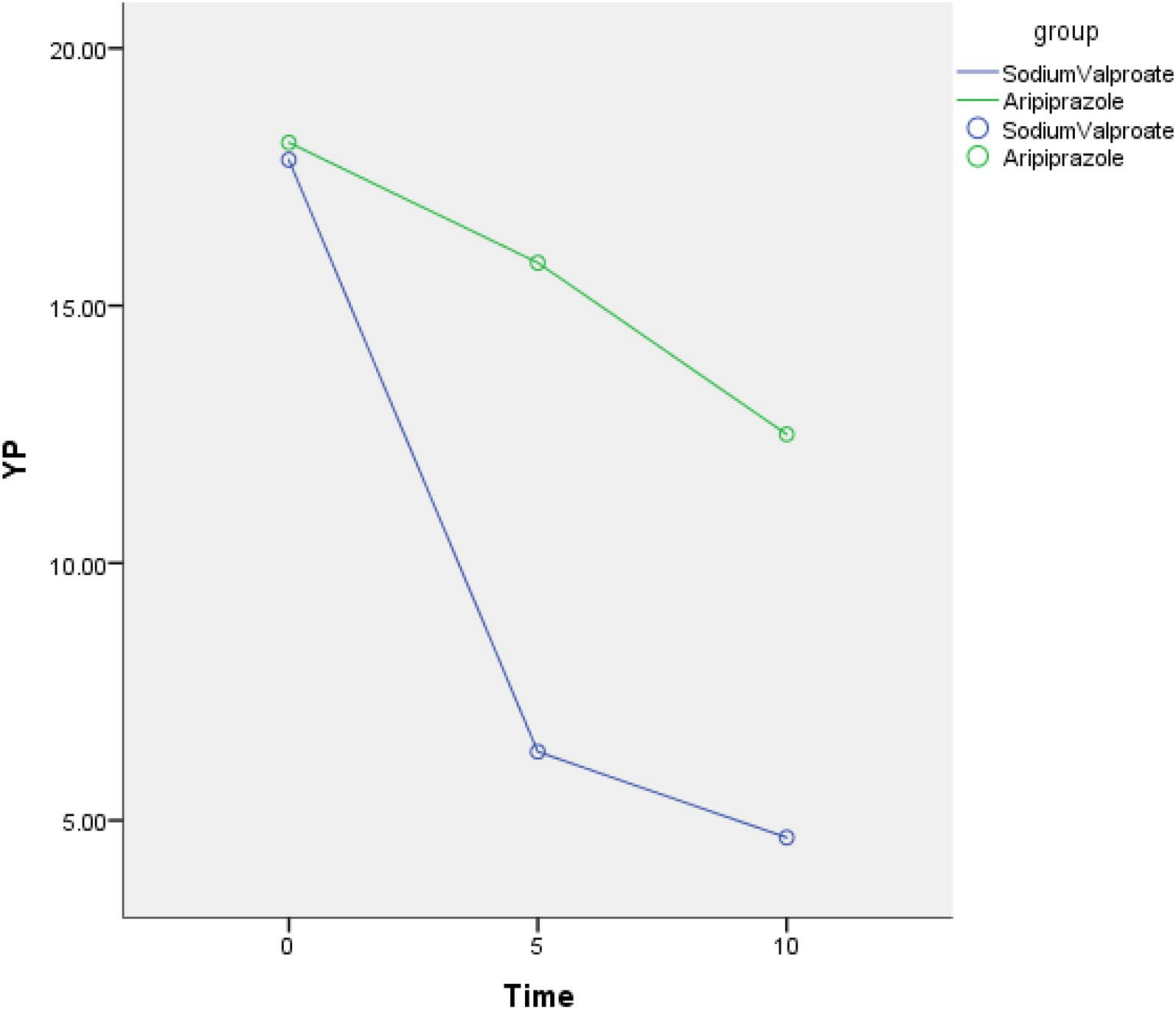
The mean YGTSS total phonic scores from baseline to endpoint

**Fig. 4:**
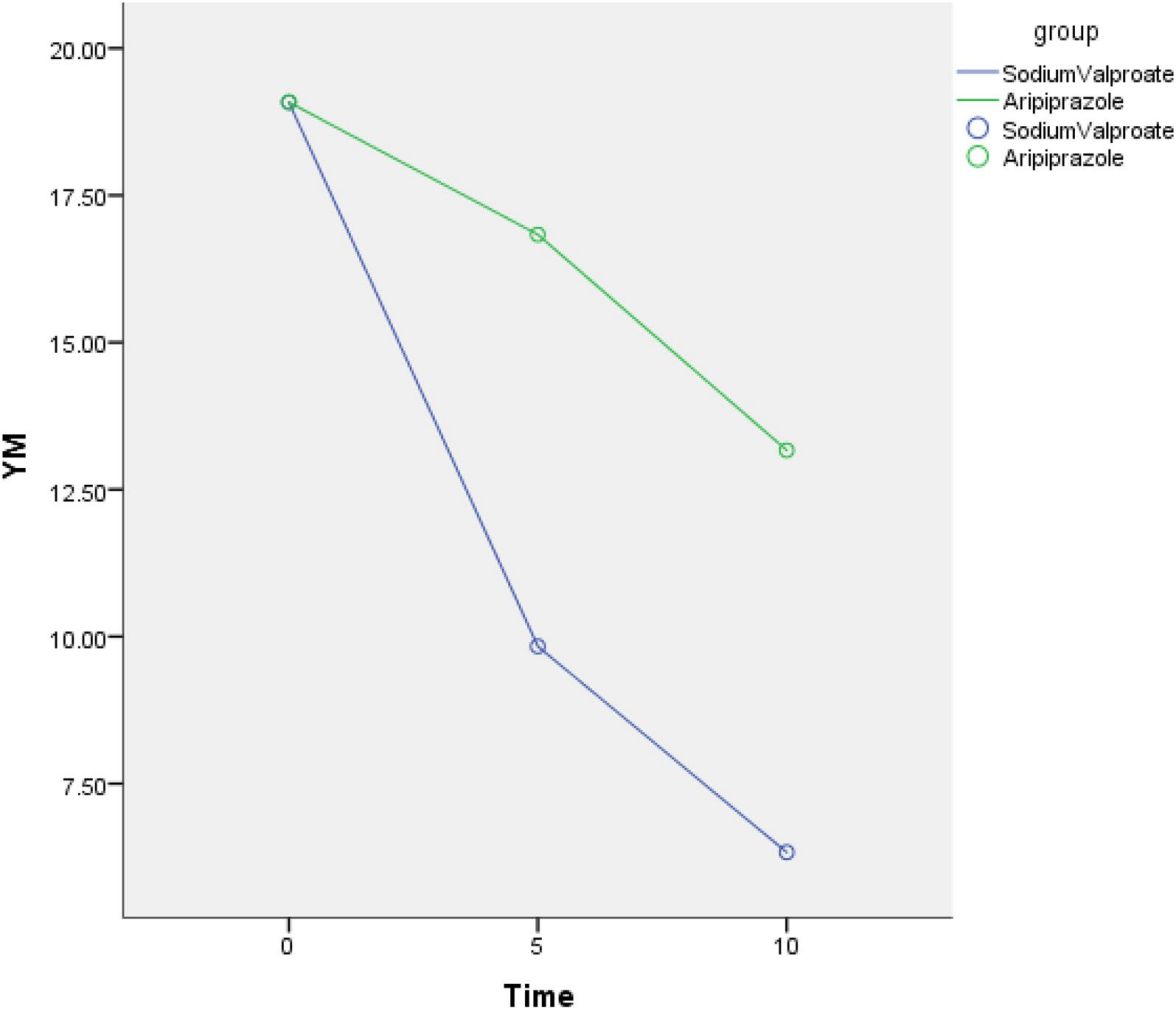
The mean YGTSS motor Tic scores from baseline to endpoint

At baseline there were no significant between-group differences in tic scores, but at each follow-up visit, tic scores including total tic scores (YT) [Fig. 1], total motor scores (YM) [Fig. 4] total phonic scores (YP) [Fig. 3] and impairment scale scores (YI) [Fig. 2] decreased over time in both groups indicating that these two drugs were efficacious in reducing tic symptoms (Table 2). Patients with severe TS had mean (±SD) pretreatment (baseline) YGTSS Global Severity score (33.33 ± 4.92) declined significantly to end point (2.50 ± 4.52; *p* < 0.001) while YGTSS Total Tic scores declined significantly from baseline (36.92 ± 5.16) to end point (11.00 ± 3.36; *p* < 0.001)) in the sodium valproate group. Patients had mean (±SD) pretreatment (baseline) YGTSS Global Severity score (34.17 ± 5.15) declined significantly to end point (20.00 ± 4.26; *p* < 0.001) while YGTSS Total Tic scores also declined significantly from baseline (37.25 ± 4.77) to end point (25.67 ± 4.62; *p* < 0.001) in the aripiprazole group. This reduction was particularly marked at the first follow-up visit (5th day) in both groups and was sustained throughout the study period. At the first follow-up visit there has significant differences between-group with YGTSS Total Tic scores and YGTSS Global Severity score. There were significant between-group effects or interactions (Table 2).

**Table 2:** Efficacy of sodium valproate and aripiprazole in the treatment of TS

	Sodium valproate			Aripiprazole			Group		Time		Group × time	
	Baseline	5 days	10 days	Baseline	5 days	10 days	*F*	*p*	*F*	*p*	*F*	*p*
YM	19.08 (2.23)	9.83 (1.53)	6.33 (1.23)	19.08 (2.19)	16.83 (1.99)	13.17 (2.48)	64.907	< 0.001	177.651	< 0.001	31.961	< 0.001
YV	17.83 (3.33)	6.33 (2.54)	4.67 (3.28)	18.17(2.76)	15.83 (3.04)	12.50 (2.43)	95.256	< 0.001	23.863	< 0.001	46.152	< 0.001
YF	33.33 (4.92)	11.67 (3.89)	2.50 (4.52)	34.17(5.15)	25.83 (5.15)	20.00 (4.26)	198.000	< 0.001	62.663	< 0.001	29.333	< 0.001
YT	36.92 (5.16)	16.17 (3.10)	11.00 (3.36)	37.25(4.77)	32.67 (4.98)	25.67 (4.62)	164.455	< 0.001	63.035	< 0.001	35.209	< 0.001

### Efficacy of aripiprazole and sodium valproate: clinical global impression

Ten (83.33%) subjects achieved CGI-Tic Improvement scores of 1 (‘‘very much improved’’) or 2 (‘‘much improved’’) in the sodium valproate group while seven (58.33%) subjects achieved CGI-Tic Improvement scores of 2 (‘‘much improved’’) in the aripiprazole group at the first follow-up visit so there was a statistically significant difference between the sodium valproate group and the aripiprazole group (*Z* = − 2.705, *p* = 0.008). Although the tics score decreased during the treatment in both groups, the patient's self-evaluation or psychological improvement of the disease in the sodium valproate group was better than in the aripiprazole group. The CGI-I scores of both groups decreased over time (*Z* = − 2.705, *p* = 0.008) without significant between-group effects or interaction effects (Table 3).

**Table 3:** The rate of adverse effects by the groups

	Time (days)	Sodium valproate (n = 12)	Aripiprazole (n = 12)	*Z*	*p*
CGI-I	5	1.00 (1.00, 2.00)	2.00 (2.00, 3.00)	− 2.705	0.008
	10	1.00 (1.00, 1.00)	2.00 (2.00, 2.00)	− 3.104	0.004
TESS	5	0.00 (0.00, 1.00)	0.00 (0.00, 1.00)	− 0.173	0.887
	10	1.00 (0.25, 1.75)	1.00 (0.25, 1.00)	− 0.619	0.590

### The Treatment Emergent Symptom Scale

Most common adverse effects were mild which included nausea (4 patients), drowsiness (4 patients), decreased appetite (1 patient), and sensitivity (1 patient) in the sodium valproate group emerged at the beginning while getting better with time.

Adverse effects in the aripiprazole group emerged when the dose was increased in an attempt to target symptoms that did not respond to lower dosage included hypersomnia (4 patient), xerostomia (2 patient), decreased appetite (1 patient), decreased sleep (1 patient), and tremor (1 patient). At the two follow-up visits, there was no significant difference in adverse events between groups (Table 3).

## Discussion

Patients having continuous phonic tics or motor tics almost all the day had severe TS, which seriously affects the daily life and mental health of patients and the impairment scale scores is extremely high. The total tic scores was above 35 and the impairment scale scores was above 30 in this experiment, indicating that the child was in a serious state of persistence at the time of treatment. The tics had a serious impact on the daily life and psychology of the children and their family members, so it is necessary to find a way to reduce the tic symptoms quickly, effectively and safely. Oral drug aripiprazole has a unique mechanism of action which needs a period for 4–8 weeks or even more longer time in controlling tics. A 10-week multicenter, double-blind, randomized, placebo-controlled trial on 61 children and adolescents with the diagnosis of Tourette’s disorder showed that aripiprazole in comparison to placebo was effective and relatively safe in the short-term treatment [23]. Another study indicates that oral aripiprazole is a safe and effective treatment for tics in children and adolescents with Tourette’s disorder in 8 weeks [15]. Not only the complexity of tic disorders, but also the effectiveness and adverse effects of the medications are major challenges for us so we chose to start at a dose of (2.5 mg/day < 50 kg, 5 mg/day ≥ 50 kg) before bedtime; patients had no bad reaction after oral administration. On the 5th day, the patient still had obvious tic symptoms, and the average dose of 2.71 mg/day was significantly lower than the average dose reported in the literature, so we adjusted to (5 mg/day < 50 kg, 7.5 mg/day ≥ 50 kg).The average dose after 5 days was 5.2 mg/day, which was lower than the average dose of previous children and adolescents, but the patients had some discomfort including lethargy (4 cases, 33.3%) and xerostomia (2 cases, 16.67%), decreased appetite (1 case, 8.3%), decreased sleep (1 case, 8.3%), and tremor (1 case, 8.3%) [Table 4]. The adverse effects mainly occurred after the addition of treatment and the incidence of adverse effects is lower than the previous literature, which does not rule out that our observation time is short or the therapeutic dose is lower than normal. Sodium valproate (VPA) controls twitching by modulating the central GABA system and upregulating EAAT1 mRNA and protein levels. Intravenous valproic acid does not require organic solvent to dissolve, can be injected at physiological pH, minimizes the risk of reaction at the injection site, the incidence of adverse events is low, and the effect is stable. In the experiment, we chose the dose 30 mg/kg/day, once every 12 h. Adverse reactions in the experiment mainly occur in the early stage of treatment and with the treatment going on, most adverse reactions disappear and does not affect normal life at all (Fig. 2).

Current results of this randomized, open-label, control clinical trial suggest that although the two groups are all effective in the reduction of tics in children and adolescents with TS, the sodium valproate group became more lower than the aripiprazole group on the day 5, and the patient's impairment scores was also significantly lower than in the aripiprazole group. Although the family were happy with the tics scores reduction, there were still concerns about whether the condition would repeat again. The average age of the patients we selected was 6–16 years, with an average of 9–10 years. Parents lead the children to visit many medical institutions in Beijing and Shanghai, and repeatedly carry out various examinations. The cost is huge. Some parents refuse to take regular treatment, choose to continue observation and cause the patient's condition to increase year by year. Parents are under tremendous economic and psychological pressure during the process. Due to the special pathogenesis of TS, the anxiety of parents may affect the treatment of children, and some even seriously affect the patient's condition. The family are very concerned about the speed of treatment and they need a quickly way to control tics. Some patients have self-injury behaviors due to exercise tics, the body and the psychology were all under great pressure who were also concerned about if there is a way in controlling tics quickly and safely in a short time (Fig. 3).

Our results showed that the CGI-I score of the sodium valproate group was significantly better than the aripiprazole group on the 5th day. The difference between the two groups was statistically significant (*Z* = − 2.705, *p* = 0.008). It showed that with the tics scores decreased, the patients improved their self-evaluation or psychological improvement. The sodium valproate group was better than the aripiprazole group. On the 10th day, the CGI-I score of the sodium valproate group was significantly better than the aripiprazole group. The difference between the two groups was statistically significant (*Z* = − 3.104, *p* = 0.004). Although aripiprazole is widely used in the treatment of tic disorder, and parents are still worried about antipsychotic drug adverse effects. There was no significant difference between the CGI scores in 5 days and 10 days (*p* > 0.05) in sodium valproate group which indicates that the patient's self-evaluation has not changed significantly with the progress of the treatment over time, but the TESS score between 5 days and 10 days has statistically significant difference (*p* < 0.05) in the sodium valproate group. The patient indicated that the symptoms are obviously improved after 5 days of treatment, but that the improvement was not as obvious as before after 10 days of treatment and the clinical improvement did not reach the expectations of the family. Increasing the days of treatment did not significantly improve the patient's impairment score while the side effects increased (Table 4), so we recommend the treatment of sodium valproate injection for 5–7 days (Fig. 4).

Because the patients selected in this study are in a relatively serious state of tic disorder, the incidence of this condition is low in the clinic. Although our department is the main medical institution for treating tic disorder in the eastern part of Heilongjiang Province, its representativeness is still limited so we hope to cooperate with more hospitals to expand the sample size in the future (Table 4).

**Table 4:** The rate of adverse effects by the groups

Adverse events	Sodium valproate* N* = 12 (* N*, %)	Aripiprazole* N* = 12 (*N*, %)
Drowsiness	4, 33.3%	0
Nausea	4, 33.3%	0
Xerostomia	0	2, 16.7%
Decreased sleep	0	1, 8.3%
Decreased appetite	0	1, 8.3%
Hypersomnia	0	4, 33.3%
Sensitive	1, 8.3%	0
Tremor	0	1, 8.3%

## Conclusions

The study indicates that the patients treated with sodium valproate injection have a faster onset time than the patients treated with oral aripiprazole in controlling tics. Because of the limited number of patients in our studies, we hope to cooperate with more hospitals in China to expand the sample size in the future.

## Data Availability

All data generated or analyzed during this study are included in this published article.

## Abbreviations

YGTSS: Yale Global Tic Severity Scale
EEG: electroencephalograph
MRI: magnetic resonance imaging
ECG: electrocardiograph
OD: once daily
GABA: gamma aminobutyric acid
NE: norepinephrinergic
DA: dopaminergic
